# A Multi-Modal Approach to Islet and Pancreas Transplantation With Calcineurin-Sparing Immunosuppression Maintains Long-Term Insulin Independence in Patients With Type I Diabetes

**DOI:** 10.3389/ti.2023.11367

**Published:** 2023-06-08

**Authors:** Steven A. Wisel, Andrew M. Posselt, Gregory L. Szot, Miguel Nunez, Keli Santos-Parker, James M. Gardner, Giulia Worner, Garrett R. Roll, Shareef Syed, Yvonne Kelly, Casey Ward, Medhi Tavakol, Kristina Johnson, Umesh Masharani, Peter G. Stock

**Affiliations:** ^1^ Comprehensive Transplant Center, Cedars-Sinai Medical Center, Los Angeles, CA, United States; ^2^ Division of Transplantation, University of California, San Francisco, San Francisco, CA, United States; ^3^ Department of Surgery, University of California, San Francisco, San Francisco, CA, United States; ^4^ Division of Endocrinology, University of California, San Francisco, San Francisco, CA, United States

**Keywords:** immunosuppression, immune tolerance, insulin independence, islet transplant, pancreas transplant

## Abstract

Long-term success in beta-cell replacement remains limited by the toxic effects of calcineurin inhibitors (CNI) on beta-cells and renal function. We report a multi-modal approach including islet and pancreas-after-islet (PAI) transplant utilizing calcineurin-sparing immunosuppression. Ten consecutive non-uremic patients with Type 1 diabetes underwent islet transplant with immunosuppression based on belatacept (BELA; *n* = 5) or efalizumab (EFA; *n* = 5). Following islet failure, patients were considered for repeat islet infusion and/or PAI transplant. 70% of patients (four EFA, three BELA) maintained insulin independence at 10 years post-islet transplant, including four patients receiving a single islet infusion and three patients undergoing PAI transplant. 60% remain insulin independent at mean follow-up of 13.3 ± 1.1 years, including one patient 9 years after discontinuing all immunosuppression for adverse events, suggesting operational tolerance. All patients who underwent repeat islet transplant experienced graft failure. Overall, patients demonstrated preserved renal function, with a mild decrease in GFR from 76.5 ± 23.1 mL/min to 50.2 ± 27.1 mL/min (*p* = 0.192). Patients undergoing PAI showed the greatest degree of renal impairment following initiation of CNI (56% ± 18.7% decrease in GFR). In our series, repeat islet transplant is ineffective at maintaining long-term insulin independence. PAI results in durable insulin independence but is associated with impaired renal function secondary to CNI dependence.

## Graphical Abstract

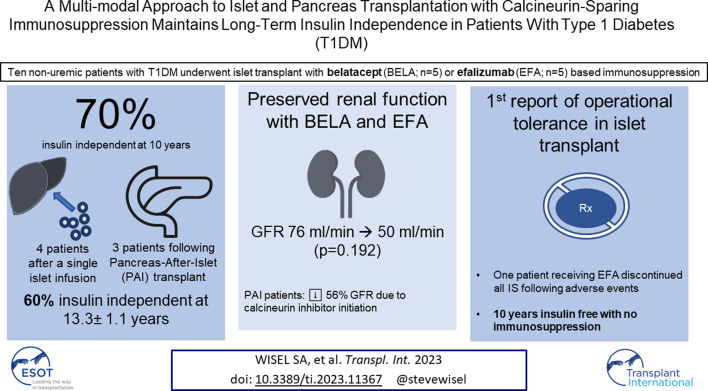

## Introduction

Over five million Americans are expected to be living with Type 1 diabetes by 2050 [1]. For most patients, insulin therapy remains the mainstay of management to control blood glucose and minimize microvascular complications [2, 3]. Continuous glucose monitors, wearable insulin pumps, and mobile applications have improved patient compliance and outcomes in managing Type 1 diabetes with exogenous insulin [3, 4]. However, beta cell replacement—either by solid organ pancreas transplant or islet transplantation—remains the sole curative intervention for Type 1 diabetes and avoids the life-threatening complication of hypoglycemic episodes associated with intensive insulin therapy [5–9]. Long-term islet function beyond 5 years remains a challenge, and patients may require additional islet infusions to restore insulin independence. Previous studies from our group and others have shown that pancreas-after-islet (PAI) and islet-after-pancreas (IAP) transplantation provide additional multimodal pathways to maintain long-term insulin independence for patients with Type 1 diabetes [10–12].

Choice of immunosuppression remains a critical factor in maintaining long-term beta cell function and optimizing patient outcomes. Calcineurin inhibitors (CNI) have revolutionized the field of transplantation, but the known beta cell and renal toxicities limit their efficacy for pancreas and islet transplantation. Our group has previously published 5-year outcomes in a cohort of ten consecutive patients receiving CNI-sparing maintenance immunosuppressive regimens based on either the costimulation blocker belatacept (BELA; LEA29Y, BristolMyers Squibb, New York, NY) or the antileukocyte functional antigen-1 antibody efalizumab (EFA; Raptiva, Genentech, Inc., S. San Francisco, CA; JDRF grant #4-2004-372) [13–15]. BELA is a cytotoxic T-lymphocyte-associated protein-4 (CTLA-4) fusion protein which binds CD80 and CD86 on antigen-presenting cells and serves to block the co-stimulation necessary for full T cell activation after antigen-specific T cell receptor binding occurs. By blocking this crucial second-step, T cell activation is prevented, and the T cell instead undergoes anergy and apoptosis. BELA has been shown to be effective in preventing graft rejection, allowing reduced levels of conventional immunosuppressive medications, and has no beta-cell or renal toxicity [14, 16, 17]. EFA is a monoclonal antibody which binds the CD11a subunit of leukocyte function antigen-1 (LFA-1), inhibiting its binding to the intercellular adhesion molecule-1 (ICAM-1), thereby preventing adhesion of leukocytes which is a necessary step for T cell activation and trafficking [18–20]. Despite its efficacy in islet transplantation, several patients in a concurrent trial of EFA for psoriasis developed progressive multifocal leukoencephalopathy (PML) and thus EFA was withdrawn from the market in May 2009 [21].

Here, we describe 10-year outcomes in a cohort of pre-uremic patients with Type 1 diabetes who received islet transplants with BELA- and EFA-based immunosuppression. Our results detail a multi-modal, personalized approach to beta cell replacement including a combination of islet and pancreas transplant in pursuit of long-term insulin independence for Type 1 diabetes patients while limiting the adverse effects of CNI and preserving renal function. Additionally, we review the clinical course of one islet transplant recipient who continues to maintain operational tolerance 13 years after a single islet infusion, over 9 years after discontinuing all immunosuppression.

## Materials and Methods

### Patients

Patients were consecutively enrolled in this study as previously described [13]. To meet inclusion criteria for islet transplant during this study, patients had a history of Type 1 diabetes mellitus for a minimum of 5 years, baseline C-peptide level less than 0.5 ng/mL, and/or a history of symptomatic severe hypoglycemic episodes. Eligible patients were required to have a body mass index less than 30 kg/m^2^ (or weight less than 80 kg), a daily total insulin requirement below 55 units/day, and preserved renal function. Patients meeting inclusion criteria were screened for malignancy before undergoing informed consent in accordance with the institutional review board and clinical protocols registered at clinicaltrials.gov [13, 15].

During the study period, all islet transplants performed at our institution were completed as a part of this clinical trial. All 10 islet transplant were performed consecutively within the institutional experience, and no patients meeting inclusion criteria were excluded from participation in the study or transplanted under alternate immunosuppression protocols. Study enrollment was completed at time of islet offer, with patients undergoing a standardized informed consent process in addition to a research consent as approved by the institutional review board. Enrollment in the study was non-randomized: the first five patients enrolled received efalizumab and the second five patients received belatacept.

### Islet Preparation and Transplantation

Pancreatic islet isolation was performed as previously described [13, 15]. Enrolled patients received at minimum 4,000 islet equivalents (IEQ) per kg body weight, with islets having at minimum 70% viability and glucose stimulated insulin secretion index above 1.0. All islet allografts during this study met criteria, and no islet preparations were discarded post-enrollment. Islets were cultured until infusion by percutaneous transhepatic cannulation of the portal vein. Patients received systemic anticoagulation via intravenous heparin infusion for the first 48 h after transplant, followed by an additional 5 days of therapeutic enoxaparin injections subcutaneously. Patients were considered for a second infusion of islets 2–3 months after loss of insulin independence. Patients were selectively considered for subsequent pancreas-after-islet (PAI) transplant if they had returned to insulin use following at least one islet transplant, were deemed acceptable surgical candidates, and expressed willingness to proceed with pancreas transplant.

### Immunosuppression

For induction, all patients received 2 mg/kg/day of antithymocyte globulin (thymoglobulin) for 2 days (4 mg/kg total dose) prior to islet transplantation, with one dose of methylprednisolone prior to the first thymoglobulin administration. The BELA and EFA immunosuppression regimens have previously been described [13]. Patients enrolled in the BELA protocol (*n* = 5) received 10 mg/kg intravenously on days 0, 4, 14, 28, 56, and 75 post-transplant, followed by 5 mg/kg every 4 weeks until 18 months post-transplant, followed by 5 mg/kg every 8 weeks thereafter. Patients receiving the EFA immunosuppression protocol (*n* = 5) received 1 mg/kg subcutaneously every week from 1 day prior to transplant until 3 months post-transplant, followed by 0.5 mg/kg per week. Immunosuppression was supplemented with sirolimus and mycophenolic acid (MPA) as previously described [13].

Following FDA discontinuation of EFA in May 2009, three of the EFA-treated patients were continued on combination immunosuppression with sirolimus and MPA, one patient was continued on MPA monotherapy, and one patient was continued on combination therapy with tacrolimus and MPA.

Immunosuppression for patients receiving PAI transplantation has been previously described [12]. Briefly, PAI recipients received induction therapy with anti-thymocyte globulin (6 mg/kg) and methylprednisolone. Patients were then started on a three-drug maintenance immunosuppression regimen including tacrolimus (trough target 5–7 ng/mL), mycophenolic acid (360–720 mg twice daily) and prednisone 5 mg per day, with addition of a mammalian target of rapamycin (mTOR) inhibitor (sirolimus or everolimus) at 1-month post-transplant. A full summary of patient immunosuppression is available in Supplementary Table S1.

### Post-Transplant Patient Monitoring

Patients were asked to perform blood glucose checks three to five times daily, with accurate recording of both dietary intake of carbohydrates and use of insulin. Patients underwent serial laboratories for fasting serum blood glucose, Hemoglobin A1c (HbA1c) levels, and C-peptide levels. Post-transplant HbA1c levels above 6.4% were considered hyperglycemic and defined loss of insulin independence. Partial insulin use was defined as less than 0.5 units/kg/day, with full insulin use defined as greater than 0.5 units/kg/day. Patient renal function was also closely monitored to evaluate for glomerular filtration rate (GFR). All patients undergoing PAI transplantation were subject to protocol pancreas biopsies between 2- and 6-month post-transplant as long as they were medically stable to undergo the procedure.

### Immunologic Screening and Alloreactive T cell Frequency Analysis

Patient whole blood samples were collected pre-transplant and at pre-determined post-transplant intervals (days 7, 14, 28, 56, 75, 90, 120, 175, 270, and 365). Specimens were processed by Ficoll gradient to isolate peripheral blood mononuclear cells (PBMC) for immunologic analysis. As previously described, specimens were cryopreserved in liquid nitrogen at −196°C prior to flow cytometric analysis for phenotypic characterization by expression of CD3, CD4, CD8, CD25, Foxp3, and CD127 (13, 15).

Patient PBMCs were evaluated for alloreactivity *in vitro* as previously described [13]. Patient PBMC, donor splenocytes, and third-party donor splenocytes were thawed from cryopreservation and resuspended in complete RPMI (RPMI 1640 supplemented with human AB serum (heat-inactivated), 100 U/mL penicillin, 100 μg/mL streptomycin, and 2 mM sodium glutamate) [15]. Patient PBMCs were cultured alone, with third-party splenocytes, or with donor splenocytes. *Staphylococcus* enterotoxin B (1 μg/mL) was used as a positive control in separate culture with patient PBMCs. Cultures were maintained for 5 days, at which point brefeldin A (Epicentre, 10 μg/mL) was added to culture for 6 h. Cells were harvested, washed with PBS twice, and FACSPerm Solution II (BD Biosciences) was used to permeabilize cells for 10 min. Cells were labeled with antibodies to CD4, CD8, PerCP, IFN-γ, TNF-α, APC, and IL-4 (BD Biosciences).

### Statistical Analysis

Data are presented as means ± standard error except where otherwise stipulated. A student’s t-test was used for statistical analysis, with a *p*-value of less than or equal to 0.05 as significant. For analysis of renal function in patients with preserved islet function, individual patient GFR data was utilized. For data only recorded as creatinine (mg/dL) or normal GFR (>60 mL/min), estimated GFR values were computed using the CKD-EPI Creatinine Equation (2021). Renal function (GFR) for each individual patient was modeled over time using restricted cubic splines, utilizing 4 knots per patient. The model-based values allowed alignment in approximate 1 month intervals so that mean profiles using all subjects in each group could be computed. The mean ± standard error GFR profiles are reported for each group. Computations were carried out using R 4.0.5 (R Foundation for Statistical Computing, Vienna, Austria. https://www.R-project.org).

## Results

### Donor and Recipient Characteristics

Islet and pancreas donor characteristics have previously been described [12, 13], with recipient characteristics summarized in Table 1. A total of 14 islet transplants were performed into 10 recipients, with four patients receiving a second infusion of islets. The number of IEQ administered per islet transplant ranged from 402,666 to 691,500 IEQ (mean 575,131 ± 92,923 IEQ), corresponding to a dose of 6,101 to 12,825 IEQ/kg (mean 9,554 ± 1,980 IEQ/kg).

**TABLE 1:** Donor and recipient characteristics for islet and pancreas-after-islet transplant.

Recipient	Age at Islet Txp	Gender	BMI	Preislet HbA1c	Islet Txp 1			Islet Txp 2			PAI Txp			Total Follow Up (mos.)
					Total IEQ	IEQ/kg	Duration of Insulin Independence (mos.)	Total IEQ	IEQ/kg	Duration of Insulin Independence (mos.)	Age at PAI Txp	Time between Islet and PAI Txp (yr.)	PRA at PAI Txp	
BELA-1	56	F	24.7	8.7	507,660	7,577	143							144
BELA-2	53	F	23.6	6.5	645,500	10,940	144							144
BELA-3	60	F	19.1	7.6	691,500	12,805	54							147
BELA-4	43	M	29.3	8.4	608,400	8,112	19	724,125	9655	13	48	2.9	2%	153
BELA-5	62	F	21.2	7.6	557,500	9,450	10	450,000	8215	62				156
EFA-1	47	F	25.4	6.7	542,777	12,825	1	573,128	9244	82				167
EFA-2	58	F	21.3	8.2	661,409	10,667	169							169
EFA-3	48	F	25.6	7.3	482,050	8,000	27				51	3.2	10%	172
EFA-4	58	F	19.2	8	630,165	11,458	172							172
EFA-5	40	F	24.9	6.7	577,950	8,711	4	402,666	6101	25	44	3	62%	177

Recipients were between 40 and 62 years old at time of their first islet transplant. Nine out of ten patients included in this study were female, with BMI ranging from 19.1 to 29.3 kg/m^2^ (mean 23.4 ± 3.2 kg/m^2^). All recipients were confirmed to have diabetes, with pre-transplant HbA1c values of 6.7%–8.7% (mean 7.6% ± 0.8%). Baseline GFR measured from 41 mL/min to 98 mL/min (mean 76.5 ± 23.1 mL/min). Patients were followed for an average of 4,714 ± 399 days following their first islet transplant (12.9 years; range 4,214–5,286 days). As previously described, patients received EFA for a range of 392–804 days prior to drug removal from market and transition of immunosuppression [13].

Three patients went on to PAI transplant following loss of islet function. Two patients (BELA-4 and EFA-5) had previously received two islet infusions, while one patient (EFA-3) expressed an interest to proceed to pancreas transplant following a single islet transplant. Patients undergoing PAI transplant had a panel reactive antibody (PRA) of 2%–62% with a mean interval of 3.0 ± 0.15 years between final islet infusion and subsequent pancreas transplant.

### Post-Transplant Islet and Pancreas-After-Islet (PAI) Graft Function

Four patients maintained long-term insulin independence following a single islet infusion for an average duration of 157 ± 15.3 months (13 years; range 144–172 months); two of these patients received BELA-based immunosuppression (BELA-1 and BELA-2) and two of these patients received EFA-based immunosuppression (EFA-2 and EFA-4). Three of these patients remain insulin independent, while the fourth patient (BELA-1) resumed partial insulin dependence 11.8 years after islet transplant.

**FIGURE 1:**
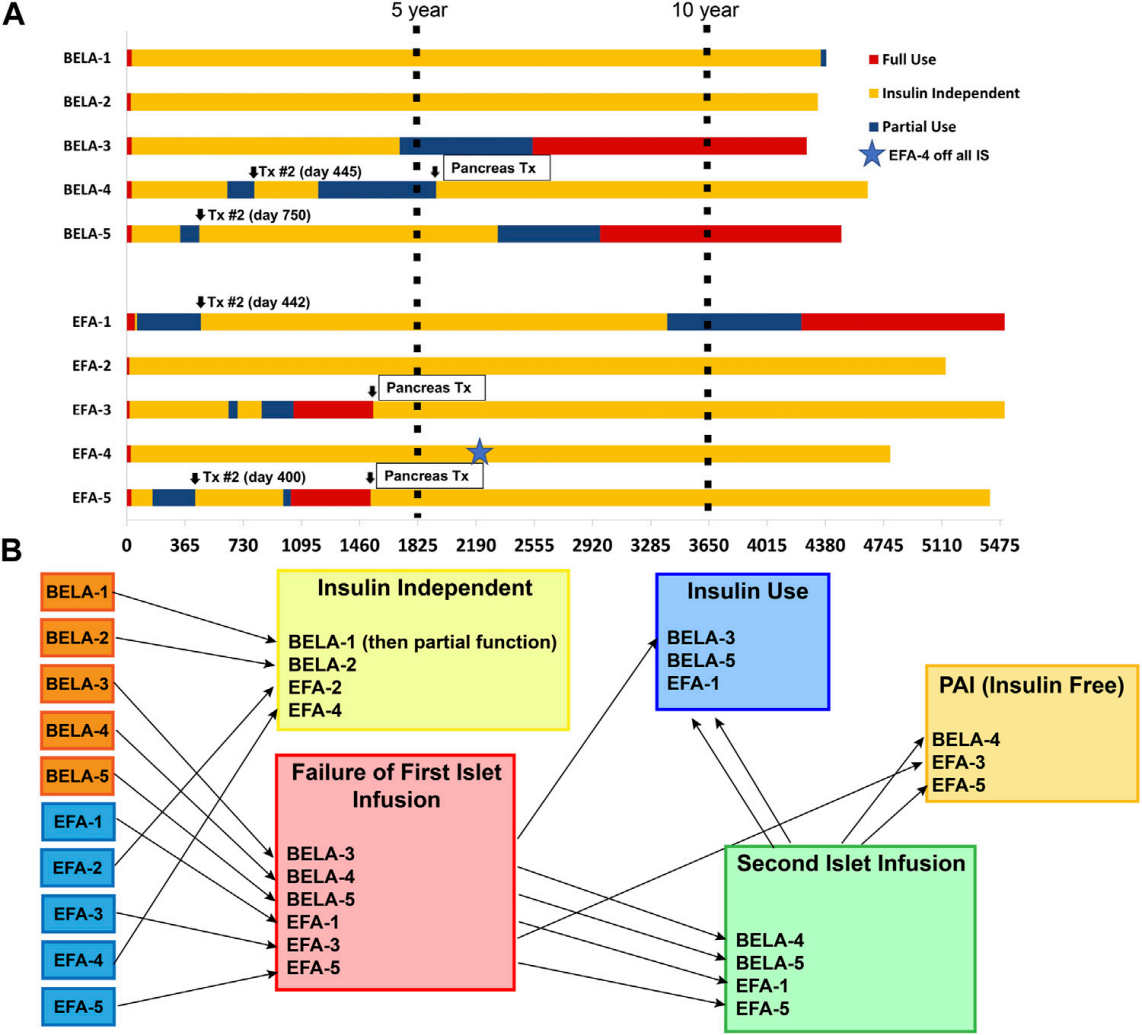
Islet and Pancreas-After-Islet (PAI) outcomes for patients receiving CNI-sparing immunosuppression based on Belatacept (BELA) or Efalizumab (EFA). **(A)** Summary of beta cell replacement outcomes from time of transplant; **(B)** Summary of beta cell replacement outcomes by graft function status.

Six patients experienced failure of their first islet transplant after an average of 19 ± 19 months (range 1–54 months). Four of these patients (BELA-4, BELA-5, EFA-1, and EFA-5) proceeded with a second islet infusion, one patient (BELA-3) elected to forego a second islet infusion after 54 months of insulin independence and returned to insulin use, and one patient (EFA-3) proceeded to PAI transplant after returning to insulin use 27 months post-islet infusion. For patients undergoing a second islet transplant, the mean duration of insulin independence was 45.5 ± 32.0 months (range 13–82 months), with all four returning to insulin use following a second islet infusion. Two of these four patients (BELA-4 and EFA-5) underwent PAI transplant following failure of their second islet transplant, while the remaining two patients (BELA-5 and EFA-1) remain on exogenous insulin therapy. At time of publication, six out of ten patients continue to experience insulin independence (two of five BELA patients and four of five EFA patients), including the three patients who underwent PAI transplantation. Islet and PAI outcomes are summarized in Table 2; Figure 1.

**TABLE 2:** Recipient outcomes, immunosuppression, and adverse events following islet and pancreas-after-islet transplant.

Recipient	Initial HbA1c	Current HbA1c	Current Insulin	Initial GFR	Current GFR	Duration of EFA (days)	Current IS	Complications	Management	PAI Transplant	
										Rejection	Treatment
BELA-1	7.4	7.3	6 U Tresiba morning	98	91		Sirolimus/myfortic/ belacept	Oral mucosal lesions			
BELA-2	5.9	6	None	44	48		Myfortic/belacept	EBV viremia, subnephrotic proteinuria	stopped sirolimus, proteinuria improved		
BELA-3	6.9	7.4	10 U Tresiba morning, Aspart 2-3 U with meals (∼16–19 U/day)	80	54		None	Proteinuria, nonhealing leg wound in 2013	Stop sirolimus, increase myfortic		
BELA-4	8	5.7	None	75	26		Tac/Everolimus/ Myfortic/ Prednisone	Bronchitis	Antibiotic therapy	No	N/A
BELA-5	7.1	6.1	14 U determir at night, 2–3 U insulin regular with meals (∼20–23 U/day)	41	8		None	CMV esophagitis; cryptococcal meningitis	valgancyclovir, amphotericin/ flucytosine -> chronic fluconazole		
EFA-1	6.9	6.7	Lantus 10–12 U BID, humalog 28 –30 U (∼ 49–53 U/day)	91	99	504	None	None	N/A		
EFA-2	6.8	6.1	None	50	41	568	Sirolimus/myfortic	Intraperiotneal bleed; Thrush; UTI	None; antifungal therapy; antibiotic therapy		
EFA-3	7.6	5.6	None	91	45	583	Tac/Myfortic/ Prednisone	Peripancreatic abscess (PAI); Gastrointestinal distress, insomnia (PAI)	Percutaneous drainage, antibiotic therapy; dicsontinuation of mTOR inhibitor	Grade 1 ACR	Thymoglobulin, methylprednisolone
EFA-4	6.1	5.9	None	100	44	392	None	Angioinvasive aspergillosis of the lung; CMV viremia; PTLD	Voriconazole, withdrawal of mTOR; valganyclovir and IS dose reduction; complete IS withdrawal, rituximab		
EFA-5	6.7	5.1	None	95	46	804	Tac/Myfortic/ Prednisone	Portal vein thrombus (islet); Facial squamous cell carcinoma (PAI)	Anticoagulation; Local resection	No	N/A

### Post-Transplant Glycemic Control

**FIGURE 2:**
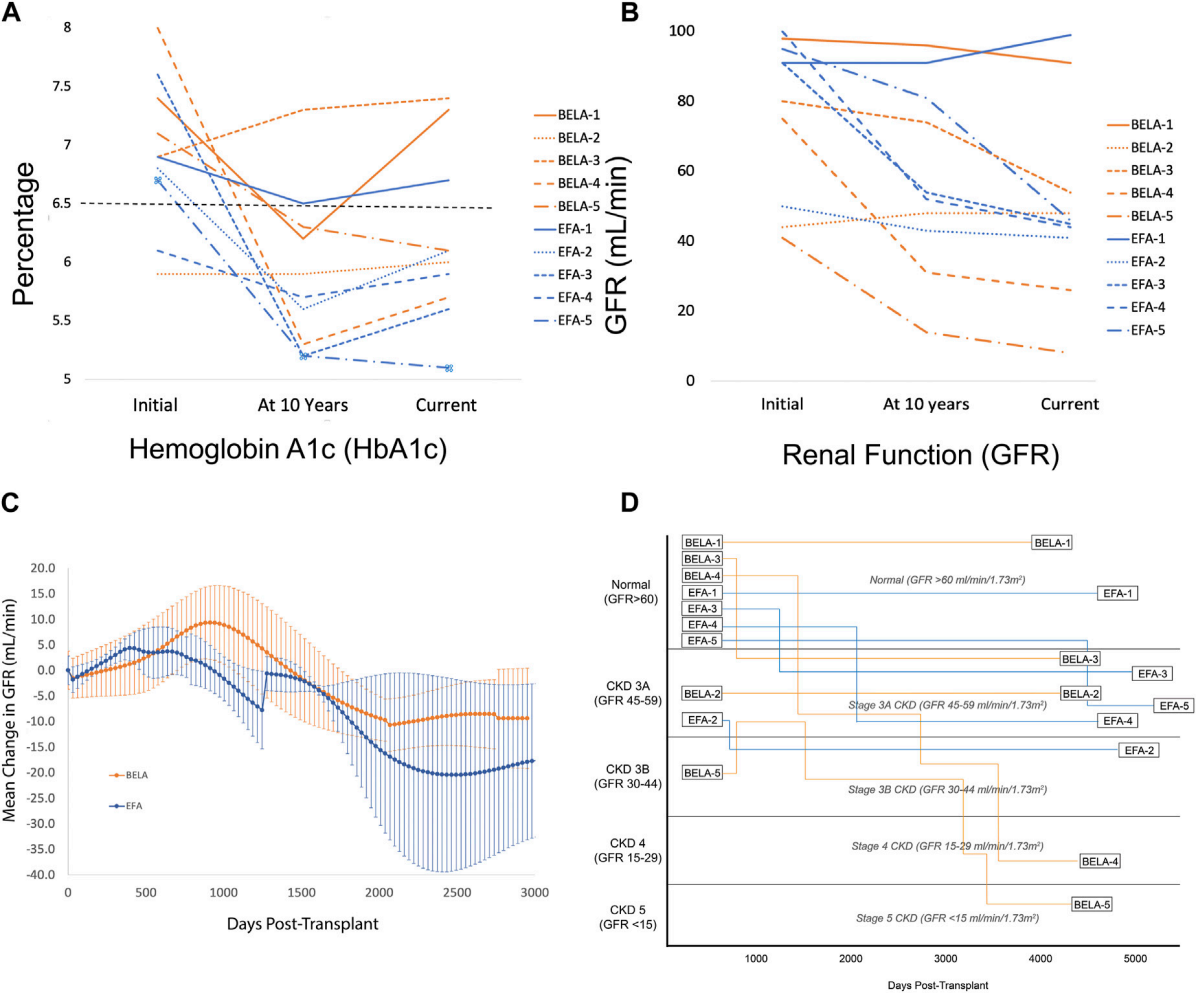
**(A)** Glycemic control measured by HbA1c (%); **(B)** Renal function measured by GFR (mL/min); **(C)** Change in GFR for patients with preserved islet function; **(D)** Renal function by GFR and stage of kidney disease from time of transplant.

Three of five BELA patients and four of five EFA patients remained insulin independent at 10 years, with improvement in HbA1c from 7.1% ± 1.1% to 5.8% ± 0.45% for BELA patients and 6.8% ± 0.6% to 5.5% ± 0.4% for EFA patients (Figure 2A). All patients were free from hypoglycemic unawareness, irrespective of insulin independence. Beyond 10 years, one patient (BELA-1) resumed partial insulin use 11.8 years post-transplant, while six patients remain insulin independent with average follow-up of 4,867 ± 384 days from first islet infusion (13.3 ± 1.1 years, range 4,307–5,286). The four patients reliant on exogenous insulin use (BELA-1, BELA-3, BELA-5, and EFA-1) require a range of 6–53 regular insulin unit equivalents per day (Table 2).

### Preservation of Renal Function

Overall, patients demonstrated preserved renal function with a mild decrease in GFR from 76.5 ± 23.1 mL/min to 50.2 ± 27.1 mL/min over the duration of the study (*p* = 0.192; Figure 2B). Patients receiving BELA demonstrated slight decrease in GFR from 67.6 ± 24.5 mL/min to 45.4 ± 31.4 mL/min (*p* = 0.077), and patients receiving EFA demonstrated mild downtrend in GFR from 85.4 ± 20.1 mL/min to 55.0 ± 24.7 mL/min (*p* = 0.073). When analyzing patients with functional islet grafts, renal function was preserved for both BELA (−9.4 ± 9.8 mL/min) and EFA (−17.8 ± 15.1 mL/min) patients, with no significant difference between BELA and EFA cohorts (Figure 2C). The three patients undergoing PAI transplant, with initiation of a CNI-based immunosuppression regimen, demonstrated the highest degrees of renal impairment over the study interval, with a mean decrease in GFR, from 92.6 ± 16.4 mL/min at time of PAI transplant to 39 ± 11.3 mL/min (−56% ± 18.7%) at most recent follow-up. Overall, eight of ten patients maintained renal function over the course of the study, with two patients progressing to stage 4 or stage 5 CKD (Figure 2D). One patient (BELA-4) progressed to stage 4 chronic kidney disease (CKD) following PAI transplant, while one patient (BELA-5) progressed from stage 3B to stage 5 CKD following islet transplant failure.

### Adverse Events

A total of 19 complications occurred in 10 patients over the study period and are summarized in Table 2. Following islet transplant, one patient (EFA-2) experienced post-infusion bleeding while another (EFA-5) experienced a partial portal vein thrombus which resolved following a course of oral anticoagulation. Three patients had complications related to sirolimus administration while additional infectious complications included bronchitis, oral thrush, and urinary tract infection. Complications following PAI included peri-pancreatic abscess requiring drainage and antibiotic therapy (EFA-3), squamous cell carcinoma requiring local resection (EFA-5), and bronchitis which resolved following antibiotic therapy (BELA-3). Two patients (BELA-5 and EFA-4) developed severe opportunistic infections, including CMV esophagitis, cryptococcal meningitis, pulmonary aspergillosis, and post-transplant lymphoproliferative disorder (PTLD). Infectious complications responded to antiviral and antifungal medications, and immunosuppression was completely withdrawn with rituximab administration for treatment of PTLD in patient EFA-4.

### Post-Transplant Immunologic Screening

**FIGURE 3:**
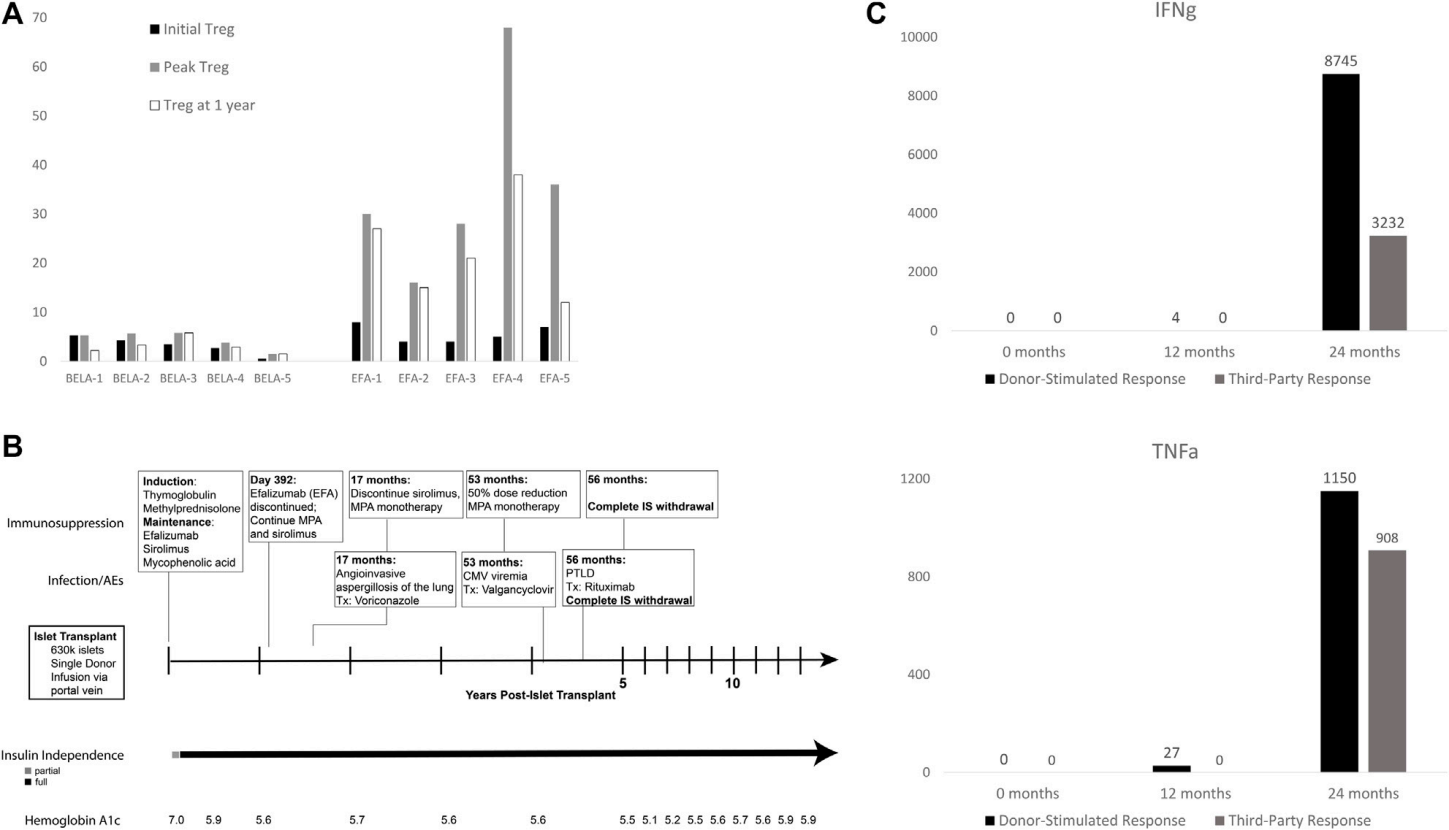
**(A)** Treg expansion in first year post-transplant (adapted from Posselt, et. al., 2010); **(B)** Operational tolerance in patient EFA-4 following withdrawal of immunosuppression; **(C)** EFA4 cytokine production in mixed lymphocyte reaction to donor-specific (black) and third-party (gray) antigen. EFA4 demonstrated no reactivity to donor or third-party antigen in the first 12 months post-transplant, which returned by 24 months.

The early effects of BELA and EFA on circulating levels of CD4^+^ FoxP3+ regulatory T cells (Treg) in the first year following islet transplant have previously been reported along with medium-term outcomes in this cohort, but remain relevant to patient outcomes (Figure 3A) [13, 15]. Briefly, patients receiving BELA demonstrated stable levels of Treg as a percentage of total circulating T cells in the first year post-transplant, with Tregs comprising 3.3% ± 1.8% of circulating T cells at time of transplant, a peak level of 4.4% ± 1.8% of circulating T cells, and 3.1% ± 1.6% at 1 year post-transplant. EFA patients demonstrated increased levels of circulating Treg in the first year following islet transplant, with Treg prevalence of 5.6% ± 1.8% at time of transplant (*p* = 0.076), peak Treg percentages of 35.6% ± 19.5% (*p* = 0.0074), and Tregs comprising 22.6% ± 10.4% of the circulating T cell population at 1 year post-transplant (*p* = 0.0032).

### A Case of Operational Tolerance in Islet Transplant Recipient

As described above, patient EFA-4 demonstrated evidence of profound over-immunosuppression, characterized by multiple post-transplant adverse events (Figure 3B). At 17 months post-transplant, the patient was diagnosed with angioinvasive aspergillosis of the lung, prompting treatment with voriconazole and reduction of immunosuppression to MPA monotherapy. The patient developed CMV viremia at 53 months post-transplant which was cleared following valganciclovir and MPA dose reduction to 360 mg BID. However, biopsy done at 56 months post-transplant for fatigue and gingival soreness confirmed diagnosis of post-transplant lymphoproliferative disorder (PTLD). All immunosuppression was withdrawn and the patient was treated with eight cycles of rituximab with complete remission. Since being removed from all immunosuppression, the patient continues to maintain insulin independence following a single islet infusion, demonstrating a case of operational tolerance in an islet transplant recipient.

Patient EFA-4 showed profound expansion of Tregs from a normal level of 5% and reaching a peak of 68% of all circulating T cells at 30 days post-transplant. The percentage of Tregs in the peripheral blood remained high for the first year post-transplant, with Tregs comprising 38% of circulating CD4^+^ T cells at 1 year. Further functional analysis by alloreactive T cell frequency assay demonstrated lack of *in vitro* proliferation with absence of interferon-gamma (IFNg) or tumor necrosis factor-alpha (TNFa) production on exposure to donor-specific and third-party antigen for the first 12 months post-transplant (Figure 3C). Following withdrawal of EFA, the patient demonstrated a return of normal cytokine production at 24 months post-transplant in response to donor-specific and third-party antigen, as compared to positive controls. Despite the return of responsiveness to allo-antigen and withdrawal of all immunosuppression for 10 years, the recipient continues to experience insulin independence without clinically significant immune response to her islet allograft, consistent with operational tolerance.

## Discussion

Beta cell transplantation requires weighing the surgical risk of operative intervention with solid organ pancreas transplant against the limited long-term durability previously demonstrated in islet transplant outcomes. Furthermore, both islet and pancreas transplantation have been limited by selection of immunosuppression, with CNI use contributing to both beta cell deterioration and chronic renal impairment [9, 22, 23]. Here, we describe a multi-modal approach using a patient-centered combination of islet and pancreas transplantation with CNI-sparing immunosuppression regimens based on BELA and EFA to achieve long-term insulin independence. Seven of ten patients maintained insulin independence at 10 years post-transplant, and six of ten patients remain insulin independent up to 14 years following a first islet infusion, with three of these patients pursuing PAI transplant as a path to insulin independence following failed islet transplant. Importantly, this series represents 10 consecutive patients, reinforcing the real-world applicability of this data.

Medium-term results previously published on this cohort of patients demonstrated insulin independence for all ten patients at 1–3 years post-islet infusion [15]. Here, we demonstrate that islet transplantation using CNI-sparing immunosuppression regimens based on BELA or EFA result in long-term insulin independence for 40% of the patients after a single islet infusion. Part of the long-term success experienced in this cohort may be attributable to the avoidance of CNI-based immunosuppression. Avoidance of CNI provides the dual benefit for this patient population of avoiding both the nephrotoxic and the beta-cell toxic effects of CNI [9, 22, 23]. Patients who maintained islet function showed no significant decrease in GFR over the course of the study, with no difference in outcomes for BELA and EFA cohorts. Two patients did experience significant decline in GFR over the course of this study. One patient progressed to stage 5 CKD following early islet failure in the setting of full insulin dependence and progression of Type 1 diabetes. A second patient progressed to stage 4 CKD following initiation of CNI at time of PAI transplant. Indeed, all three patients undergoing PAI demonstrated decline in GFR following initiation of CNI-based immunosuppression, but remain off dialysis with preserved renal function. For non-uremic patients pursuing PAI as a pathway to insulin independence, the risk of subsequent renal impairment should be emphasized, along with utilization of a multi-drug immunosuppression regimen that limits dependence on CNI.

In this study, patients initiated on both BELA and EFA demonstrated excellent long-term results for insulin independence. For patients in the EFA cohort, it is important to acknowledge that EFA was removed from market per the manufacturer’s preference following several episodes of PML identified in a simultaneous trial for psoriasis at much higher doses. Although patients received EFA for the first 392–804 days following their first islet infusion, EFA remains of interest in islet transplantation because of its early and dramatic upregulation of circulating peripheral Tregs as a percentage of total CD4^+^ cells. In one patient, operational tolerance was achieved following a profound expansion of Tregs, at one point comprising 68% of all circulating CD4^+^ T cells. This patient was ultimately discontinued off all immunosuppression due to infectious complications and PTLD, suggesting that such high levels of Tregs may predispose to an anergic state. However, the predominance of Tregs induced by EFA also may have contributed to a tolerogenic milieu, as this patient subsequently achieved operational tolerance. Pre-clinical studies have likewise demonstrated that anti-LFA monoclonal antibodies induce donor-specific tolerance in a murine model of cardiac transplant [24] and suppression of both CD4^+^ and CD8^+^ activity [25]. These findings support a re-evaluation of EFA at lower doses for use in islet transplant recipients and emphasize the significance of early EFA use at the time of transplant. In the absence of EFA, BELA remains an excellent immunosuppressive choice for islet transplant, as it shares the benefit of avoiding nephrotoxicity and beta-cell toxicity.

When considering options for beta cell replacement, the results of this cohort suggest that islet transplant may be considered as an initial transplant option with selection of an appropriate immunosuppressive agent. Once an islet transplant has failed, the next steps to re-establish insulin independence should be individualized to the patient. Importantly, all ten patients included in this study were free of hypoglycemic episodes for the duration of follow-up. The four patients who underwent a second islet infusion experienced medium-term loss of insulin independence, at an average of 1–6 years post islet transplant. In comparison, all three patients who underwent PAI transplant remain insulin independent 7–11 years post-PAI. PAI transplantation has been previously explored by our group as a pathway to re-establish insulin independence with excellent graft survival and preservation of renal function, without effect of prior immunologic sensitization on outcomes [12]. Following a failed islet transplant, we recommend PAI transplant for patients who desire a return to insulin independence if the recipient’s surgical and cardiovascular risks are deemed acceptable. For patients who cannot tolerate a major abdominal operation, additional islet infusions remain the sole route to insulin independence.

Although the rates of infectious and malignant complications related to excess immunosuppression were overall low in this study, close post-transplant infectious surveillance for all beta cell replacement patients remains essential to long-term outcomes. In addition to the adverse events described here, our series of PAI patients were at higher risk for subsequent BK virus infections [12]. Patients undergoing beta cell replacement—particularly PAI patients—are exposed to high levels of immunosuppression to maintain graft function. Given this requirement, patients should undergo vigilant post-transplant monitoring to minimize both infectious and neoplastic events.

This study is limited by the small cohort size and the non-randomized assignment of immunosuppression regimens. However, the long-term results for these patients suggest several routes of further investigation in islet transplantation. Future directions will be defined by the availability of EFA as a choice for immunosuppression, and progress on FDA approval of islet transplant as a therapeutic modality for Type 1 diabetes. Ultimately, a multi-modal approach incorporating islet and pancreas transplant with all available immunosuppressive options will maximize the benefits of beta cell replacement for patients with Type 1 diabetes mellitus.

## Data Availability

The original contributions presented in the study are included in the article/Supplementary Material, further inquiries can be directed to the corresponding author.

## Supplementary Material

Supplementary Table S1 Full summary of immunosuppression regimens for EFA and BELA patients.

## Abbreviations

BELA, belatacept; CKD, chronic kidney disease; CNI, calcineurin inhibitors; CTLA-4, cytotoxic T-lymphocyte-associated protein-4; EFA, Efalizumab; GFR, glomerular filtration rate; HbA1c, hemoglobin A1c; IAP, Islet-after-pancreas transplantation; ICAM-1, intercellular adhesion molecule-1; IEQ, islet equivalents; LFA-1, leukocyte function antigen-1; MPA, mycophenolic acid; mTOR, mammalian target of rapamycin; PAI, Pancreas-after-islet transplantation; PBMC, peripheral blood mononuclear cells; PML, progressive multifocal leukoencephalopathy; PRA, panel reactive antibody; Treg, regulatory T cells.
